# P-537. Circulation of Respiratory Viruses from Air and Human Specimens in a Pre-kindergarten-12th Grade School District in Kansas City, MO

**DOI:** 10.1093/ofid/ofaf695.752

**Published:** 2026-01-11

**Authors:** Nicole Neeley, Brian R Lee, Brittney Fritschmann, Luke C Gard, Nibha Sagar, Dithi Banerjee, Anjana Sasidharan, Olivia Almendares, Hannah L Kirking, Rangaraj Selvarangan, Jennifer Goldman, Jennifer E Schuster

**Affiliations:** Childrens Mercy Hospital, Kansas City, MO; Children's Mercy Kansas City, Kansas City, Missouri; Children's Mercy Hospital, Kansas City, Missouri; Children's Mercy Hospital-Kansas City, Kansas City, Missouri; Children's Mercy hospital, Kansas City, Missouri; Children's Mercy Hospital, Kansas City, Missouri; Childrens Mercy Hospital, Kansas City, MO; Centers for Disease Control and Prevention, Atlanta, Georgia; Coronavirus and Other Respiratory Viruses Division, National Center for Immunization and Respiratory Diseases, CDC, Atlanta, GA; Children’s Mercy Hospital, Kansas City, Missouri; Children's Mercy Hospital, Kansas City, Missouri; Children's Mercy Kansas City, Kansas City, Missouri

## Abstract

**Background:**

Air sampling with respiratory virus testing in community settings may be a potential non-invasive, scalable method to monitor respiratory viruses. We performed an ecological analysis to describe the correlation between respiratory virus detections in air and human samples across all schools to evaluate the potential for air sampling as a tool for community-level surveillance.

**Table.**
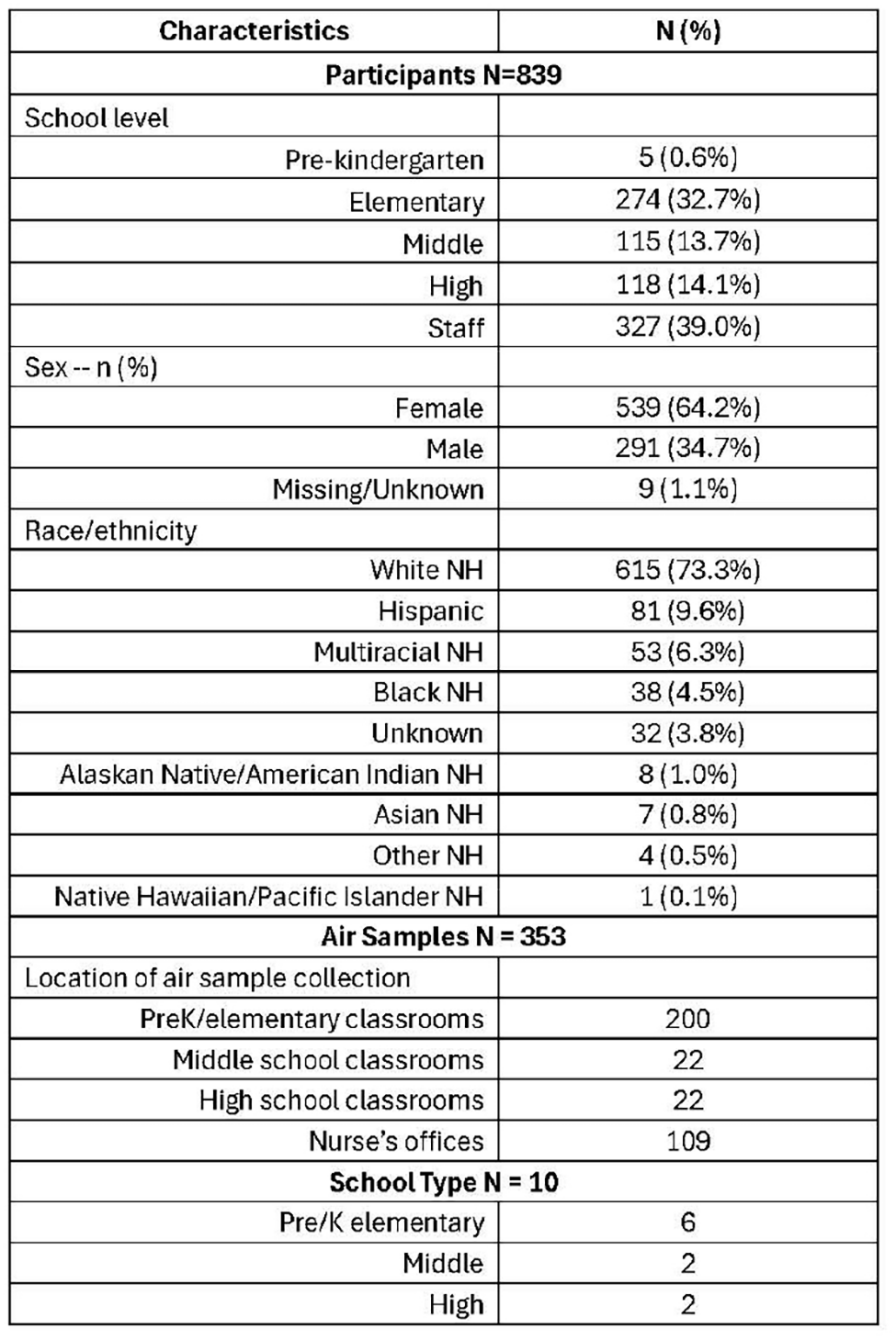
Demographics of School KIDS Student and Staff Participants Contributing ≥1 Nasal Specimen during the 2024-2025 School Year

**Figure.**
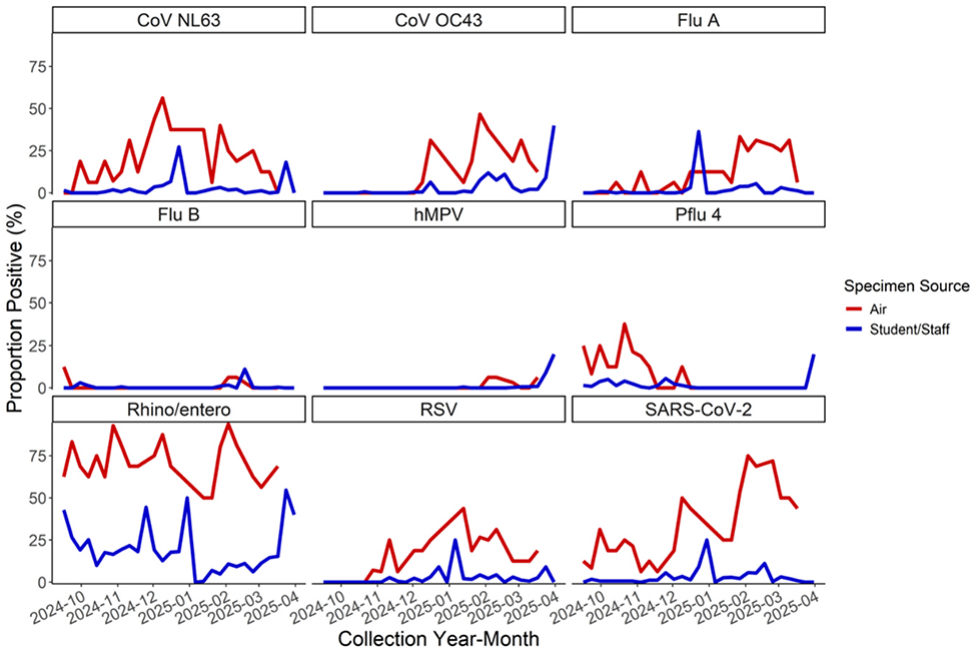
Respiratory Viruses Detected in School KIDS Pre-kindergarten-12th Grade Students and Staff Nasal and Air Specimens during the 2024-2025 School Year

**Methods:**

Knowledge of Infectious Diseases in Schools (School KIDS) is a voluntary respiratory virus surveillance program in a pre-kindergarten-12^th^ grade public school district in Kansas City, MO. In 10 schools, students and staff collected anterior nasal swabs while at school, with optional on-demand swabbing when experiencing acute respiratory illness. Simultaneously, air samples were collected biweekly using AerosolSense samplers (ThermoFisher Scientific) in 2-4 (total n=32) locations (e.g., classrooms and nurse’s office) per school for 6-8 hours during school. All specimens were tested by multiplex PCR for adenovirus, human metapneumovirus (hMPV), influenza (Flu) A and B, parainfluenza viruses (PIV) 1-4, respiratory syncytial virus (RSV), rhinovirus/enterovirus (RV/EV), SARS-CoV-2, and seasonal coronaviruses ([sCoV] OC43, HKU1, NL63, 229E). Positivity was defined as detection of ≥1 virus. District-wide weekly virus positivity rates from human and air samples were compared using Spearman’s rank correlation.

**Results:**

During September 18, 2024–March 31, 2025, 839 participants (Table) submitted 3851 specimens, with 970 (25.2%) testing positive. Of 353 air samples, 312 (88.4%) were positive. Air sample positivity was higher than human specimen positivity in all 22 weeks when both human and air samples were collected (Figure). Strong positive correlations were observed for RSV (0.87), sCoV OC43 (0.87), PIV-4 (0.73); moderate for sCoV NL63 (0.61), Flu A (0.58), and SARS-CoV-2 (0.53); and weak for hMPV (0.29) and RV/EV (0.12).

**Conclusion:**

Air sampling in schools demonstrated moderate-to-strong correlation with human respiratory virus detections for several viruses, suggesting possible utility for non-invasive public health monitoring. Further studies are needed to evaluate the relationship between air and human virus detection.

**Disclosures:**

Brian R. Lee, PhD, MPH, Merck: Grant/Research Support Rangaraj Selvarangan, PhD, Altona: Grant/Research Support|Biomerieux: Advisor/Consultant|Biomerieux: Grant/Research Support|Biomerieux: Honoraria|Cepheid: Grant/Research Support|Hologic: Grant/Research Support|Hologic: Honoraria|Meridian: Grant/Research Support|Qiagen: Grant/Research Support

